# Nevus Sebaceous of Jadassohn in Adults—Can Reflectance Confocal Microscopy Detect Malignant Transformation?

**DOI:** 10.3390/diagnostics13081480

**Published:** 2023-04-20

**Authors:** Vlad Mihai Voiculescu, Ana Maria Celarel, Elena Codruta Cozma, Madalina Laura Banciu, Mihai Lupu

**Affiliations:** 1 Department of Dermatology, Panduri Medical Center, 011367 Bucharest, Romania; 2 Department of Dermatology, “Carol Davila” University of Medicine and Pharmacy, 050474 Bucharest, Romania; 3 Department of Dermatology and Allergology, Elias Emergency University Hospital, 011461 Bucharest, Romania; 4 Department of Pathophysiology, University of Medicine and Pharmacy of Craiova, 200638 Craiova, Romania

**Keywords:** confocal microscopy, nevus sebaceous of Jadassohn, basal cell carcinoma, dermoscopy, skin

## Abstract

Nevus sebaceous of Jadassohn (NSJ) is a rare congenital lesion that affects the adnexal structures of the skin. It is typically located on the scalp and face of females and presents as a well-defined, slightly elevated, yellow lesion. It is also linked to a high risk of secondary tumors, which are more frequently benign than malignant. In vivo reflectance confocal microscopy (RCM) is a non-invasive imaging technique that provides a horizontal image of the skin with a resolution similar to histology. We report a case of basal cell carcinoma (BCC) developed in an NSJ with its dermoscopic, confocal, and histopathological features. A 49-year-old female presented with a well-circumscribed, 1 cm-diameter verrucous, yellowish lesion surrounded by a poorly defined, slightly erythematous, translucent plaque, located on the scalp in the temporoparietal region, which had been present since birth, grew at puberty, and changed its appearance in the last three years. Dermoscopy of the central lesion revealed yellow globules grouped into clusters, with peripheral linear and arborescent thin vessels, surrounded by several translucent nodular lesions with fine, arborizing vessels. RCM examination showed large, monomorphic cells with a hyperreflective periphery and a hyperreflective center located on the central lesion, corresponding to sebocytes, surrounded by multiple dark silhouettes lined with hyperreflective bands of thickened collagen, corresponding to tumor islands. The histopathological findings confirmed the diagnosis of BCC developed on an NJS. RCM can be a useful technique for the non-invasive examination and monitoring of these lesions, taking into account their transformation risk and preventing unnecessary excisions that might have a detrimental aesthetic impact on patients.

## 1. Introduction

Nevus sebaceous of Jadassohn (NSJ) is an organoid nevus that involves the epidermis, the pilosebaceous unit, and apocrine glands [1]. These congenital lesions might be present at birth or emerge during infancy, affecting 0.3% of children with a similar incidence rate among both genders [2,3]. The scalp and the face are the most common sites affected by sebaceous nevus. It manifests clinically as a well-defined, slightly raised, yellow lesion with a soft surface. Alopecia might be associated with its location on the scalp. This lesion can expand during adolescence and acquire a cerebriform appearance [1].

In adulthood, NSJ is associated with an increased risk of secondary benign, and more often, malignant tumors [4,5]. Trichoblastoma (TB) and syringocystadenoma papilliferum are the most common benign tumors, but NSJ can also be associated with trichilemmoma, sebaceous adenoma, desmoplastic trichilemmoma, apocrine adenoma, or poroma [6,7]. Trichoblastoma is a benign skin tumor that develops from follicular germinative cells and can appear alone or in conjunction with NSJ. It typically develops on the face, neck, or scalp of individuals between the ages of 40 and 50 as a well-circumscribed papule between 5 mm and 8 cm in diameter that can be either skin color or blue-black. Its cause is unknown. It has been associated with a large number of mutations and can be linked to various genetic disorders in familial forms [8]. Syringocystadenoma papilliferum is an uncommon benign cutaneous tumor that develops from apocrine or eccrine sweat glands. It commonly appears on the head or neck of children, although it can also arise in adults. Clinically, it is a single plaque or nodule with a diameter of 1–3 cm, either alone or alongside NSJ in 30% of cases [9].

The most frequent malignant tumor is basal cell carcinoma (BCC), but other forms of carcinoma, including squamous cell carcinoma (SCC) and adnexal carcinoma, may also be present [6,7]. BCC is a common malignant tumor of the skin that develops from the cells of the basal layer or the appendages. Males are affected more frequently than females, and the prevalence rises with age. Numerous factors, including environmental ones (exposure to ultraviolet radiation), long-term immunosuppression, and genetic ones, are thought to play a role in BCC’s etiology. Clinically, BCC can manifest in a variety of ways, but often it looks like a pink papule or nodule with a transparent surface and telangiectasias located most frequently in areas exposed to the sun. However, other anatomical sites are not protected [8,10].

Clinically and dermoscopically, distinguishing between TB and BCC developed on the NSJ is difficult, especially their nodular variant. Therefore, the biopsy with histopathological examination and immunohistochemistry stains is the only one that can guide the differential diagnosis between these two entities. TB appears at histology examination as a basaloid proliferation of cells surrounded by fibrous stroma located in the dermis without an inflammatory infiltrate. On the other hand, BCC appears as a basaloid aggregate with peripheral palisading, surrounded by myxoid stroma that originates from the epidermis and retracts around the islands. A lymphocytic infiltrate is also present. Unfortunately, even histopathological evaluation cannot always distinguish between the two; thus, immunohistochemistry is useful [8,11].

In vivo reflectance confocal microscopy (RCM) is a helpful imaging method that provides a non-invasive, histological-like image of the skin, showing both individual cells and anatomical features. In dermatology, RCM can be used alongside clinical and dermoscopic examinations for a more accurate diagnosis. The in vivo evaluation of the lesions allows not only a non-invasive diagnosis but also the evaluation of the microscopic extension of the tumor margins and the guidance of a biopsy. The last one is especially useful in the case of large lesions with malignant potential in areas difficult to treat surgically, in order to establish the biopsy site more accurately. NSJ is an example of this type of tumor, in which the RCM examination before biopsy increased the probability that the biopsy taken is diagnostic for a malignant transformation [12].

This paper aims to present a BCC case developed on an NSJ on the scalp of a middle-aged female with its clinical, dermoscopic, confocal, and histopathological features, emphasizing the role of RCM in the diagnostic and management of those cases. 

## 2. Case Report

We report the case of a 49-year-old non-smoking female with a medical history of ankylosing spondylitis and osteoporosis (without immunosuppressive medication in the medical history) who presented in our dermatology clinic with a lesion on the scalp located on the temporoparietal region. This lesion had been present since birth and had increased in size during puberty. In addition, the patient noticed the lesion changing over the last three years, with the appearance of scales on the surface and localized pruritus. 

**Figure 1:**
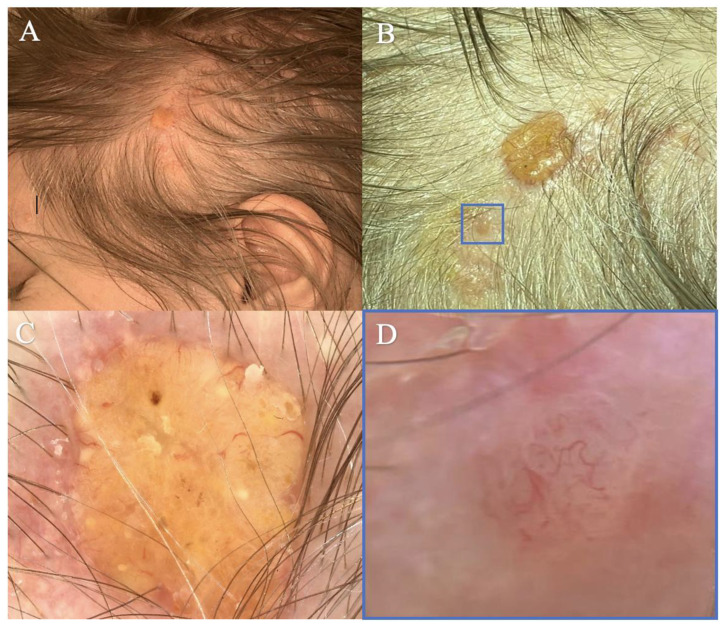
Clinical and dermoscopic aspects of the lesion. (**A**) A yellowish, verrucous lesion surrounded by a poorly defined, erythematous plaque on the scalp (temporoparietal area) of a 49 year-old female. (**B**) A close-up of the lesion from panel (**A**) (the dermoscopy of the main lesion is depicted in panel (**C**) and the dermoscopy of the lesion in the blue square is illustrated in panel (**D**)). (**C**) Dermoscopy of the central lesion showing globules aggregated in clusters and fine peripheral linear, serpiginous, and arborizing vessels. The vessels do not cross the central region of the tumor. Multiple yellow dots are observed on the surface of the lesion. (**D**) Dermoscopy of a nodular lesion in the proximity of the central one reveals a pink, poorly defined nodular lesion with fine, arborizing vessels that cross the central surface of the lesion and fade towards the periphery.

Clinical examination revealed a well-circumscribed, 1 cm-diameter verrucous, yellowish lesion surrounded by a poorly defined, slightly erythematous, translucent plaque (Figure 1A,B) located on the left temporoparietal region. Dermoscopic investigation showed yellow globules aggregated in clusters with a papillary appearance and peripheral linear and arborescent fine vessels (Figure 1C). Around this lesion, dermoscopy revealed multiple nodular lesions with a translucent appearance and fine, arborizing vessels (Figure 1D). 

**Figure 2:**
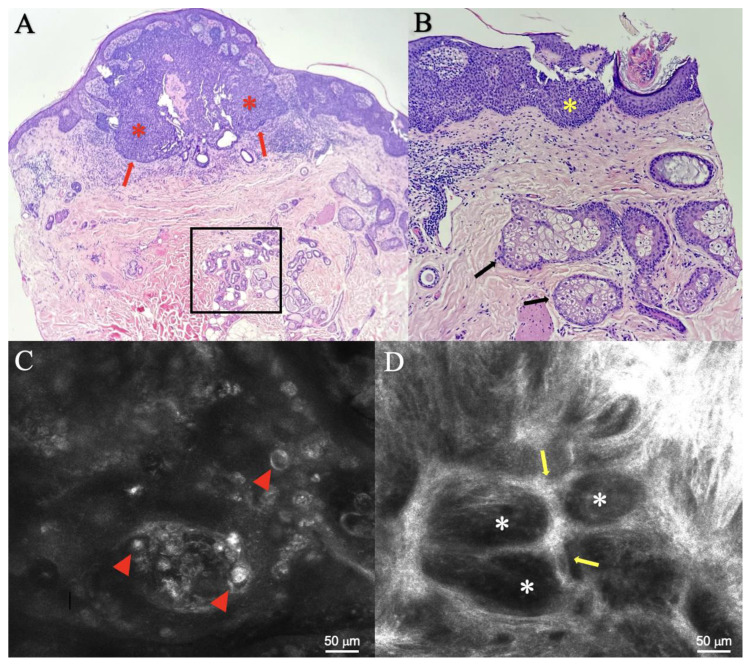
Histopathological and confocal microscopy images of the lesion. (**A**) A histopathological image showing nodular proliferation composed of islands of basaloid cells (red asterisks) with peripheral palisading (red arrows). Deep apocrine glands can also be seen (black square). (**B**) A close-up of the perilesional skin showing acanthosis (yellow asterisks) and sebaceous glands (black arrows). Hematoxylin-eosin stain, original magnification: (**A**) ×20; (**B**) ×40. (**C**) Confocal images showing large, monomorphic, hyperreflective cells with a hyperreflective center corresponding to sebocytes (red arrowheads). (**D**) Confocal microscopy image showing dark silhouettes corresponding to tumor islands (white asterisks) and hyperreflective collagen bundles surrounding tumor islands (yellow arrows).

RCM examination showed large, monomorphic cells with a hyperreflective periphery and a hyperreflective center located on the central lesion corresponding to sebocytes (Figure 2C), surrounded by multiple dark silhouettes lined with hyperreflective bands of thickened collagen located at the dermal-epidermal junction and in the upper dermis that corresponded to tumor islands (Figure 2D). 

A BCC developed on a NJS, which was suspected. Therefore, a 4 mm punch biopsy was performed with a histopathological examination that showed nodular tumor proliferation composed of islands of basaloid cells with peripheral palisading (Figure 2A) and lesional skin with hyperkeratosis, acanthosis, rudimentary pilosebaceous structures, sebaceous glands, and deep apocrine glands (Figure 2B).

The diagnosis of nodular BCC developed on an NSJ was confirmed. Following the presence of multiple confocal images in different areas of the tumor and the special localization of the lesion (temporoparietal area), surgical excision with safety margins was recommended in the plastic surgery service. As a result, the lesion was completely excised, followed by reconstruction, with favorable evolution of the postoperative scar. 

The SCALP syndrome, consisting of sebaceous nevus and other associated features such as central nervous system malformations, aplasia cutis congenita, limbal dermoid, and giant pigmented nevus, was taken into consideration [13]. However, other than the sebaceous nevus on the scalp, our patient did not exhibit any additional clinical signs that would support this diagnosis.

## 3. Discussion

NSJ is a rare congenital lesion that affects the adnexal structures of the skin. There are three different stages in the clinical progression of NSJ. The first stage occurs at birth or early childhood. It manifests as a skin color or yellow-brown plaque that can be partial alopecic, which can change appearance and color in the second stage of puberty due to hormonal influence. The third stage of development appears in adults and is associated with an increased risk of secondary benign or malignant tumors [6]. In our patient, the scalp lesion had been present from birth, had grown during the patient’s teenage years, and had changed its appearance over the past three years.

Idriss et al. [4] examined 707 cases of NSJ and discovered a secondary tumor incidence of 21.4%. TB (7.4%) and syringocystadenoma papilliferum (5.2%) were the most common benign lesions, followed by a few cases of apocrine or eccrine adenoma, trichilemmoma, and sebaceoma. Regarding the malignant tumors that appeared in 2.5% of all cases, BCC was the most frequent (5.3%), followed by SCC (2.7%), and sebaceous carcinoma (2%). These findings were consistent with those reported by Cribier et al. [5], who found TB and syringocystadenoma papilliferum as the most common benign neoplasms and BCC in 0.8% of all cases. Malignant tumors were discovered in 19% to 21% of patients, according to earlier research conducted in the 1960s. However, the high figure may be attributable to misdiagnosing TB as BCC [14].

The etiology of BCC developed on NSJ is unknown. Some articles link human papillomavirus, but this association is yet to be proven. Fitzpatrick phototypes I and II, family history, prolonged sun exposure, use of sun beds, or radiotherapy are all risk factors for BCC [15].

The development of NSJ has been linked to a mutation that occurs after zygosis in the HRAS/KRAS genes [16]. In addition, NSJ presents mutations in genes encoding PTCH and p53 that are also present in BCC. The presence of a deletion at the PTCH locus in NSJ’s basaloid cells implies the potential involvement of a tumor suppressor gene in the pathogenesis of secondary tumors, specifically BCC, in NSJ [17].

BCC and TB present similar clinical characteristics, making their differentiation challenging. Although no specific dermoscopic criteria for TB have been established, recent studies have revealed arborizing vessels in both BCC and TB, with a predominance of blue-gray ovoid nests and blue-gray globules in BCC [18,19]. In our case, the erythematous plaque and translucent nodules developed over the past three years, with dermoscopy revealing the absence of gray ovoid nests or globules along with the presence of fine arborized vessels. These clinical and dermoscopic features suggested a TB rather than a BCC, given that benign lesions are the most frequently associated with NSJ.

RCM is an effective noninvasive method for identifying BCC, with distinct characteristics reported in the literature, such as bright tumor islands, dark silhouettes, or cleft-like dark spaces [20]. However, the RCM examination has some limitations, such as a maximum imaging depth of 250 μm, which correlates with the papillary dermis, causing deeper and infiltrative tumors to be missed. Additionally, detecting distinct BCC subtypes can be difficult [21]. Currently, there are no confocal criteria for the diagnosis of TB. Pampena et al. [19] compared the confocal appearance of a pigmented TB developed on an NSJ to that of a pigmented BCC. They observed tumor islands in both instances, with peripheral clefting only seen in BCC.

From a histological perspective, TB and BCC are highly similar. Therefore, some researchers used immunohistochemistry tests with Ki-67, CK6, and 34BE12 antibodies to differentiate between BCC and TB [22].

In the case presented in this paper, at RCM examination, we observed multiple dark silhouettes surrounded by bright collagen bundles located at the dermal-epidermal junction and in the upper dermis, corresponding to tumor islands that later correlated with islands of basaloid cells with peripheral palisading at the histopathological examination, resulting in the diagnosis of BCC developed on an NSJ.

The diagnosis of NSJ is usually clinical, and the follow-up and management of these lesions are unclear due to the risk of developing malignancies. Therefore, some authors recommend closer monitoring and excision only for aesthetic purposes or in cases where malignant change is suspected, rather than prophylactic surgery in late childhood or adolescence [6]. In our case, the lesion changed its appearance in the fourth decade of the patient’s life. Therefore, after dermoscopy and RCM, we suspected a BCC had developed on an NSJ, performed a punch biopsy that confirmed the diagnosis, and excised the entire lesion with safety margins, with the patient remaining under follow-up.

Considering that NSJ can appear mainly on the scalp but also on the face, forehead, or neck, that it can be large in size, and that it can be associated with other benign or malignant lesions in its evolution [23], RCM can represent a helpful investigation in the diagnosis process, especially in cases where a biopsy or complete excision of the lesion can have a negative aesthetic impact on the patient’s appearance. VivaScope 3000, the handheld confocal microscope, is a very useful tool for evaluating large lesions in curved or difficult-to-reach areas. Furthermore, it has the ability to assess the entire lesion and its margins, guiding the location of the punch biopsy, assisting the histopathologist, or guiding the margins of the whole lesion excision. This type of examination is beneficial in areas such as the face and scalp, where reconstruction surgery can be complex [24].

## 4. Conclusions

The NSJ is a rare congenital lesion associated with the development of secondary tumors, which are more often benign than malignant. Even though dermoscopy is an excellent method for examining the NSJ, additional techniques are required for an accurate diagnosis. RCM can be a more helpful tool for the non-invasive evaluation of these lesions, examination of the microscopically extended tumor borders, and guidance of the biopsy site. RCM can also be used to monitor sebaceous nevi, considering their risk of transformation and avoiding unnecessary excisions that can have a negative cosmetic impact on patients.

## Data Availability

Not applicable.
